# Mental Power

**Published:** 1857-10

**Authors:** 


### MENTAL POWER.

REV. T. A. MILLS, one of the most practical, clear-headed, and efficient workers of our time, in his first annual report, as secretary of a permanent committee for the promotion of ministerial education, says:

"A few years ago, on a wintry morning, a boy in the habiliments of poverty entered an old school-house among our western mountains, and avowed to the master his desire for an education. There was poverty laying one of her richest gifts on the altar of religion; for that boy was JONAS KING. On his humble shoemaker's bench, CAREY laid the foundation of British Baptist Missions. John Newton found in his congregation an unfriended Scotch boy, whose soul was then glowing with new-born love to Christ. He took him to John Thornton, one of those noble merchants whose wealth, whose piety, and whose beneficence, increase together. They educated him; and that boy became CLAUDIUS BUCHANAN, whose name India will bless, when the names of Clive and Hastings are forgotten. JOHN BUNYAN was a gift of poverty to the Church. ZWINGLE came forth from an Alpine shepherd's cabin; MELANCTHON from an armorer's workshop; LUTHER from a miner's cottage; the Apostles, some of them, from fishermen's huts. These are the gifts of poverty to the Church."

Poverty compels temperance by making dietetic delicacies impossible, and allowing a very moderate supply of the necessaries of life, and even these are to be secured at the expense of physical exertion and homely work—the best tonic, the most efficient purifier of the blood ever known; hence comes a healthy body and a vigorous mind. Temperance alone does not evolve the highest grades of mental superiority; it must, in the earlier part of life, be conjoined with proportionate physical exercise. Very few of the great minds of this country have come from the city, or the cradle of the rich. The farm and the workshop have supplied by far the largest number of our eminent men. Perhaps, after all, we have never yet arrived at the true system of education. Higher results, more truth, less error, and progress without fanaticism, may result from an education conducted up to twenty-five years of age, as follows: When the child begins the tenth year, let him be impressed with the duty and necessity of "doing something for a living." Let nine o'clock be the hour for bed-time, winter and summer; leave bed at the end of eight hours, study until eleven o'clock, then dress for work during the balance of the day.

We believe that any literary man would be wiser, better, healthier, at the end of a year, by the systematic prosecution of a plan similar to the above. To rise by daylight, the year round ; by the time he is dressed it will be light enough to read. Then, in the cool and quiet of the morning, when the brain is most vigorous, let him study with all the energy he can master for four or five hours; then dress for labor, take his breakfast, and go to work, pursuing it with a steady moderation until the close of the day, that is, until sundown, then take his supper, after which, until bed-time of nine o'clock, let him employ himself in social intercourse with family and friends, and in acquainting himself with the progress of things through the newspapers and periodical press—for without this last, any man of this age will soon become a mere cypher as to influence, and an ignoramus as to acquirements. True progress *now* consists in unlearning much that is old, and in acquainting one's self with the new, in order to be able to determine its worth

